# UNITe: exploiting conserved links between oncogenesis and homeostasis to identify novel cancer drivers

**DOI:** 10.3389/fonc.2026.1711517

**Published:** 2026-02-24

**Authors:** Piyush Agrawal, Annan Timon, Vishaka Gopalan, Arashdeep Singh, Sridhar Hannenhalli

**Affiliations:** 1 Division of Medical Research, SRM Medical College Hospital & Research Centre, SRMIST, Kattankulathur, India; 2 University of Pennsylvania, Philadelphia, PA, United States; 3 Cancer Data Science Lab, National Cancer Institute, National Institutes of Health, Bethesda, MD, United States

**Keywords:** cancer driver, homeostatic processes (HP), machine learning, protein-protein interaction, regeneration, stress response, wound healing

## Abstract

**Background:**

Similarities between oncogenesis and several homeostatic processes (HPs)—wound healing, regeneration, and cellular stress response—have long been recognized. However, the molecular underpinning of these similarities is not fully understood. While several molecular aspects of HPs are evolutionarily conserved, different species exhibit substantial variation in the genes involved in these HPs, as well as in their predisposition to cancer.

**Methods:**

We leveraged 75 published (with 321 experiments across 14 species) experimental datasets of genes implicated in HPs across multiple species from Gene Expression Omnibus (GEO), pan-cancer (32 cancer types) multi-omics datasets from The Cancer Genome Atlas (TCGA), and several benchmarking datasets from public repositories [such as Genotype Tissue Expression (GTEx), MSigDB, COSMIC, and the Cancer Dependency Map (DepMap)], as well as literature to comprehensively investigate links between conserved aspects of HPs and human cancers. We performed several analyses to understand broad mechanistic links between cancer and the homeostatic processes.

**Results:**

We compiled high-confidence conserved consensus gene sets for stress response (SR), wound healing (WH), and regeneration (RG)—jointly as HPs for “homeostatic processes”. We found that broadly across cancers, the HP genes exhibit elevated mutations, including copy number aberrations and differential gene expression in the tumor compared to healthy tissue, and are associated with patient survival. In the human protein interaction network, HP genes cluster by the process type as well as with the known cancer driver genes. Leveraging this observation, here, we presented a tool, UNITe (Uncovering Network-based Interactions between Homeostatic processes and Tumorigenesis), which predicts cancer drivers based solely on network proximity to HP genes, with an area under the receiving operating characteristic (AUROC) of 0.81, far better than several current approaches, and across multiple benchmark datasets. Applying UNITe genome-wide, we reported several novel potential cancer drivers and validated them using multiple lines of evidence.

**Conclusions:**

Overall, we presented a first comparative analysis of cancer drivers with conserved homeostatic processes, suggesting a complementary approach to prioritizing cancer drivers. The model and the codes are freely available for public usage at https://github.com/hannenhalli-lab/conserved_links_homeostasis_oncogenesis.

## Introduction

Homeostasis refers to the maintenance of stable physio-chemical conditions within an organism despite dynamically varying internal and external environments (1). The key biological processes that have evolved to maintain homeostasis include stress response (SR) (1–3), wound healing (WH) (4–7), and regeneration (RG) (1, 8). Given that cancer is characterized by a breakdown and hijacking of normal homeostatic processes (5, 9, 10), links between cancer and SR, WH, and RG have been noted. For instance, WH occurs in multiple stages, including blood clotting, inflammation (11, 12), proliferation of tissue stem cells, and maturation (6). In addition to being a critical component of homeostasis, WH is also closely related to cancer. In fact, several of the same signaling pathways mediating WH, such as cell proliferation, migration, and angiogenesis, are also involved in oncogenesis (6, 13, 14). Additionally, after healing, scar tissue can lead to the development of cancer or stimulate the growth of nearby cancer cells (6). Similarly, RG, the repair or replacement of damaged or lost tissues, involves the activation of various signaling pathways and the recruitment of different cell types (11). While certain organisms, such as species of fish and amphibians, have the ability to regenerate entire limbs, mammals generally have a limited regenerative capacity (12). However, certain tissues, such as the liver, possess a higher regenerative capacity (15). Like WH, RG is also linked to cancer, as several of the same signaling pathways and molecular players involved in RG are also involved in cancer initiation and progression (16). Finally, cellular response to various stresses, such as oxidative stress, DNA damage, or nutrient deprivation, involves the activation of various signaling pathways and the upregulation of certain genes to help the cell cope with the stressors (9, 17, 18). While the cellular stress response is critical for homeostasis, the chronic activation of the stress response can contribute to cancer development (19, 20).

Given the broad mechanistic links between cancer and the homeostatic processes, we aimed to comprehensively investigate the relationships among these homeostatic processes and their relationships with cancer from multiple perspectives. This investigation required, first and foremost, a curated list of genes involved in the three homeostatic processes. Recognizing that several homeostatic processes are evolutionarily conserved, we focused specifically on the links between the conserved molecular characteristics of the homeostatic processes and human cancer.

To this end, we first curated experimental studies probing these processes across multiple species and compiled high-confidence consensus gene sets for SR, WH, and RG; we will jointly refer to these as HP, standing in for “homeostatic process”. We then investigated the extent to which these HP genes are associated with different types of cancers. Our analysis revealed several variables but strong trends across cancer types and homeostatic processes. We found that, broadly, the HP genes exhibit elevated mutations, including copy number aberrations and differential gene expression in the tumor compared to healthy tissue, and are associated with patient survival.

Protein interaction networks have been extensively exploited to reveal functional relatedness among genes (17, 21, 22). We therefore investigated the relationships among the three homeostatic processes and their relationship with known cancer driver genes in multiple protein interaction networks and found that not only do the three homeostatic processes reside in close proximity with each other, SR genes being somewhat of an outlier, but also these processes are in close proximity with cancer drivers, supporting a functional HP–cancer link. Furthermore, we developed a machine learning model, UNITe (Uncovering Network-based Interactions between Homeostatic processes and Tumorigenesis), which, based solely on network proximity of a gene to various HP gene sets, can distinguish cancer drivers from other genes [area under the receiving operating characteristic (AUROC) = 0.81] far better than several current approaches and across multiple benchmark datasets. Applying UNITe genome-wide, we reported several novel potential cancer drivers and validated them using multiple lines of evidence.

To summarize, our goal was to specifically evaluate the extent to which the three major homeostatic processes—stress response, wound healing, and regeneration—inform oncogenesis. For that reason, we focused on systematically curating from the literature gene sets involved in these three processes. Once the gene sets were curated, based on our premise that cancer drivers are likely to be functionally related to the genes involved in the basic homeostatic processes, we quantified functional relatedness based on proximity in the physical or functional gene networks, and we used those proximities to various homeostatic processes to prioritize the putative cancer drivers.

Overall, we i) provided a curated resource of genes involved in stress response, wound healing, and regeneration obtained from experimental studies across species; ii) presented a first detailed investigation of functional links between cancer and three key homeostatic processes; iii) demonstrated that network proximity from the genes involved in the three homeostatic processes can help distinguish cancer drivers; and iv) identified novel cancer driver genes as a resource.

## Methods

### Compiling genes associated with stress, wound healing, and regeneration from experimental literature

Homeostatic datasets were manually curated from Gene Expression Omnibus (GEO) (23) using the search terms “wound healing”, “regeneration”, and “stress response”. This resulted in 75 datasets spanning 321 experiments across 14 species (**Table 1**). These datasets included pre- and post-treatment gene expression profiles. For RNA-seq datasets, the expression data were analyzed using DESeq2 (version 1.0.19) (24), and for microarray datasets, a modified script running GEO2R (23) according to the standard workflow was used. The resulting p-values were adjusted for multiple testing using the Benjamini–Hochberg approach to finally obtain the up- and downregulated genes for each experiment [adjusted p-value < 0.05, absolute log fold-change (LFC) ≥ 1]. Since the experimental datasets came from several species, all differentially expressed genes (DEGs) were mapped to their closest human ortholog using the Ensembl BioMart database (25); to map bacterial genes to humans, the OMA browser was used (26). Any genes that did not have a human ortholog were excluded. To construct consensus gene sets from the DEGs of disparate experimental datasets, based on the frequency with which a gene was represented across at least two separate datasets, six gene sets—SR-UP, SR-DOWN, WH-UP, WH-DOWN, RG-UP, and RG-DOWN—jointly referred to as stress–wound–regeneration (SWR) gene sets, were constructed. In addition to SWR gene sets, gene sets were extracted from the human-centric curated databases Gene Ontology (GO) (23), Kyoto Encyclopedia of Genes and Genomes (KEGG) (27, 28), and the Molecular Signatures Database v2025.1 (MSigDB) (29) using the same search terms as our experimental dataset search (“wound healing”, “regeneration”, and “stress response”) and compiled additional consensus gene sets (SR-H, WH-H, and RG-H). Taking these together with SWR gene sets, the nine gene sets were jointly referred to as SWR+ gene sets.

**Table 1:** Curated studies pertaining to stress response, wound healing, and regeneration. The table enlists the number of datasets corresponding to stress, wound healing, and regeneration obtained from various model organisms.

Species	Stress	Wound Healing	Regeneration
A. andersoni	0	0	1
A. maculatum	0	0	1
A. mexicanum	0	0	47
C. elegans	53	0	0
C. lupis Familiaris	0	3	0
D. melanogaster	10	1	0
E. coli	19	0	0
H. sapiens	13	8	1
M. musculus	10	0	0
R. norvegicus	0	12	0
S. scrofa	0	97	0
X. laevis	0	30	0
X. tropicalis	0	0	5

### Functional enrichment and TCGA differential expression analyses

To characterize the relationship between SWR+ gene sets and cancer, we performed a series of analyses. i) We first examined the degree of overlap between SWR+ gene sets and known oncogenic [Hallmark (30), C4.CM (31), and COSMIC (32)] gene sets, and, as the control, we used GO and KEGG non-oncogenic datasets. We performed enrichment analysis comparing every gene set in the oncogenic or control gene set collection with each SWR+ gene set. For each of the nine SWR+ gene sets, we noted the fraction of gene sets in a specific collection (Hallmark, C4.CM, etc.) that significantly overlapped (p-value < 0.05) with the specific SWR+ gene set. ii) We examined the degree of overlap among SWR+ gene sets in a pairwise fashion. iii) We identified which specific oncogenic pathways (in Hallmark and CancerSEA collection) our SWR+ genes were highly enriched in. iv) We assessed the extent to which SWR+ genes were differentially expressed in various cancers. In 22 The Cancer Genome Atlas (TCGA) cancer cohorts and the corresponding normal tissue cohort from Genotype Tissue Expression (GTEx), we first obtained the differentially up- and downregulated genes (DEGs) in cancer (based on the top 10% most differentially expressed genes, Adj p-value ≤ 0.05), and then we assessed the significance of cancer DEG overlap with our SWR+ genes using Fisher’s exact test and adjusted the p-value using the Benjamini–Hochberg method.

### Deactivating alterations: mutational analysis

We downloaded publicly available simple somatic mutation (SSM) data from the TCGA (33) for 29 cancer types; for four cancer types, these data were not available. For each cancer type, we used the associated donor IDs, gene IDs, and consequence types (selecting only for the non-synonymous mutations, deactivating mutations involve Copy Number Variation (CNV) deletion, frameshift indels, and non-sense) to compute the frequency of observed disruptive mutations in genic regions across the donors. Using the cancer type-specific frequencies, we then extracted the top 5% most frequently mutated genes in a cancer and assessed their overlap with our SWR+ gene sets using Fisher’s exact test.

### CNV amplification analysis

Using TCGA CNV data from 33 different cancer types, in each cancer type, for each gene, we computed the fraction of samples in which the gene is amplified and then normalized across all genes (to control for cancer-specific CNV rates). We then examined the enrichment of the amplified genes in SWR+ gene sets using Fisher’s exact test.

### Survival analysis

Using survival data from the TCGA, we modeled the overall survival of cancer patients using the expression of each gene as a predictor variable, and age and sex as covariates in the Cox regression using the R library “survival” (34), resulting in p-values for each gene that were adjusted for multiple testing using the Benjamini–Hochberg method. We used the resulting hazard ratio to extract the top “hazardous” (HR > 1) and “protective” (HR < 1) genes for each cancer type. Based on False Discovery Rate (FDR) ≤ 0.05, we selected cancer types with at least 50 significant genes. Next, we further selected the top 1,000 hazardous (HR > 1) and protective genes (HR < 1) based on decreasing HR values if the number of significant genes was higher than 1,000. Lastly, we assessed the significance of the overlap of these two gene sets with our SWR+ gene sets using Fisher’s exact test.

### Shortest path analysis in PPI networks

To examine the relationship between SWR+ genes and genes known to be involved in oncogenic processes, we used the knowledge-based gene interaction network STRING v11.0 to calculate pairwise shortest paths. The STRING (35), an integrative database of experimental and computationally inferred protein–protein functional interactions, comprises 261,624 interactions between 19,344 proteins. We utilized NetworkX v3.6.1, a python network analysis framework, to extract the degrees of all of our genes and the shortest path library to calculate the shortest paths between nodes using Dijkstra’s algorithm. We obtained our oncogenic datasets from cancer hallmark processes curated in MSigDB and COSMIC. For each of the nine SWR+ gene sets X, and for each of the 51 hallmark (50 MSigDB and 1 COSMIC) gene sets Y, we calculated the pairwise shortest path lengths between genes in X and Y and compared them to the distribution of the pairwise shortest path lengths between degree-controlled background SWR+ gene set and hallmark set Y. Using the Wilcoxon rank sum test, we tested if genes in X were more proximal to genes in Y compared to degree-controlled background gene set.

### Predicting cancer drivers using machine learning

We trained six different machine learning models (SVM, RF, AdaBoost, Gradient Boosting, Logistic Regression, and ExtraTrees) using the scikit-learn v.1.8.0 package (36) to classify whether a gene is a cancer driver or not, using as a feature the gene’s network proximity from the nine SWR+ gene sets in the STRING database using medium confidence edges. Given a gene/protein *g*, we defined nine features where each feature represents, for each of the nine SWR+ gene sets *S*, the fraction of genes in *S* that are within a predetermined distance threshold *d* from *g*. We used a value of d = 2, as it represented the median distance between two random genes. We optimized this model on various parameters using GridSearch and fivefold cross-internal validation. We reported the average AUROC and Area Under the Precision-Recall Curve (AUPRC) from the five sets.

### Evaluation of our model with benchmark datasets

We obtained eight benchmark datasets from Shi et al. (37). These were comprised of datasets from the Cancer Gene Census, genes implicated in carcinogenesis via point mutations, high-confidence driver genes identified using the rule-based High-Confidence Drivers (HCD) approach, candidate genes predicted using mutation frequency-based methods, human orthologs of mouse cancer genes identified using insertional mutagenesis, human oncogenes manually curated from articles and a public oncogene database, and CTAT genes. However, as our machine learning (ML) model was developed using Cancer Gene Census, we excluded the two datasets, CGC and CGCpointMut, from our analysis. In total, we were left with six datasets to work with. We then evaluated the performance of our model using the AUROC and compared it to the accuracies obtained from 11 different cancer driver predictor algorithms obtained from **Supplementary Table 2** of Shi et al.: DNmax, DNsum, driverMAPS, DriverML, HotNet2, MaxMIF, WITER, MutPanning, nCOP, NetSig, and OncoIMPACT.

### Cell line-specific genetic dependency

CRISPR KO-based dependency probability scores were obtained from the Cancer Dependency Map (DepMap) website (https://depmap.org/portal/) by downloading the “CRISPR_gene_dependency.csv” dataset from the 2022 second-quarter (2022Q2) release (38). Gene dependency probability score estimates the likelihood of whether a specific gene is essential for cell survival and proliferation in a given biological context. It is a statistical metric ranging from 0 to 1. A higher score indicates higher gene dependency. DepMap contains genetic dependency data for cell lines grouped by cancer type from which they were derived. For each gene, we obtained the dependency score for each cancer type by averaging the scores for all cell lines derived from the specific cancer type. For a gene set, we then obtained the fraction of genes with a dependency probability score of ≥0.5, the recommended cutoff suggesting gene dependency. We then compared the fraction of such genes for various gene sets in a cancer-specific manner.

### Fractional analysis of novel cancer driver genes predicted using UNITe

Using the ExtraTrees machine learning model, which achieved the best performance in our cross-validation, we estimated the score for all genes in the genome and selected the top 2,000 and bottom 2,000 potential cancer driver genes, excluding the known drivers in any of the multiple benchmark sets. We performed the functional analyses of our predicted candidate cancer driver genes via multiple steps. First, we characterized the genes using several datasets, namely, our aforementioned TCGA differential expression, deactivating alterations, and survival analysis sets. Next, we evaluated the novel candidates by comparing the fractions of genes that satisfy a specific condition, such as being among the top differentially expressed, most frequently mutated, most hazardous genes, or most protective genes, with the corresponding fraction of negative controls that satisfy the same condition. We also performed gene ontology analysis for the top 2,000 genes using the clusterProfiler 4.0 (39) software with default parameters.

## Results

### Compiling genes involved in stress response, wound healing, and regeneration across species

To compile a database of genes associated with HP, we conducted a systematic search of the GEO (23), manually curating studies pertaining to the induction of cellular or organismal SR, WH, or RG (Methods). We thus compiled 75 studies spanning 321 experiments across 14 species, as summarized in **Table 1**.

From each experimental dataset, we obtained a list of differentially up- and downregulated genes (DEGs; adjusted p-value < 0.05, and LFC ≥ 1; Methods) shown in **Supplementary Table S1**. Note that these experiments were performed in different species, and therefore, each gene was mapped to its human ortholog using online resources (Methods). The multiple gene sets identified for the three homeostatic processes across species were integrated into six consensus pan-species upregulated and downregulated gene sets: SR-UP, SR-DOWN, WH-UP, WH-DOWN, RG-UP, and RG-DOWN (Methods; **Supplementary Table S2**). For brevity, we will refer to these six gene sets jointly as SWR.

**Figure 1:**
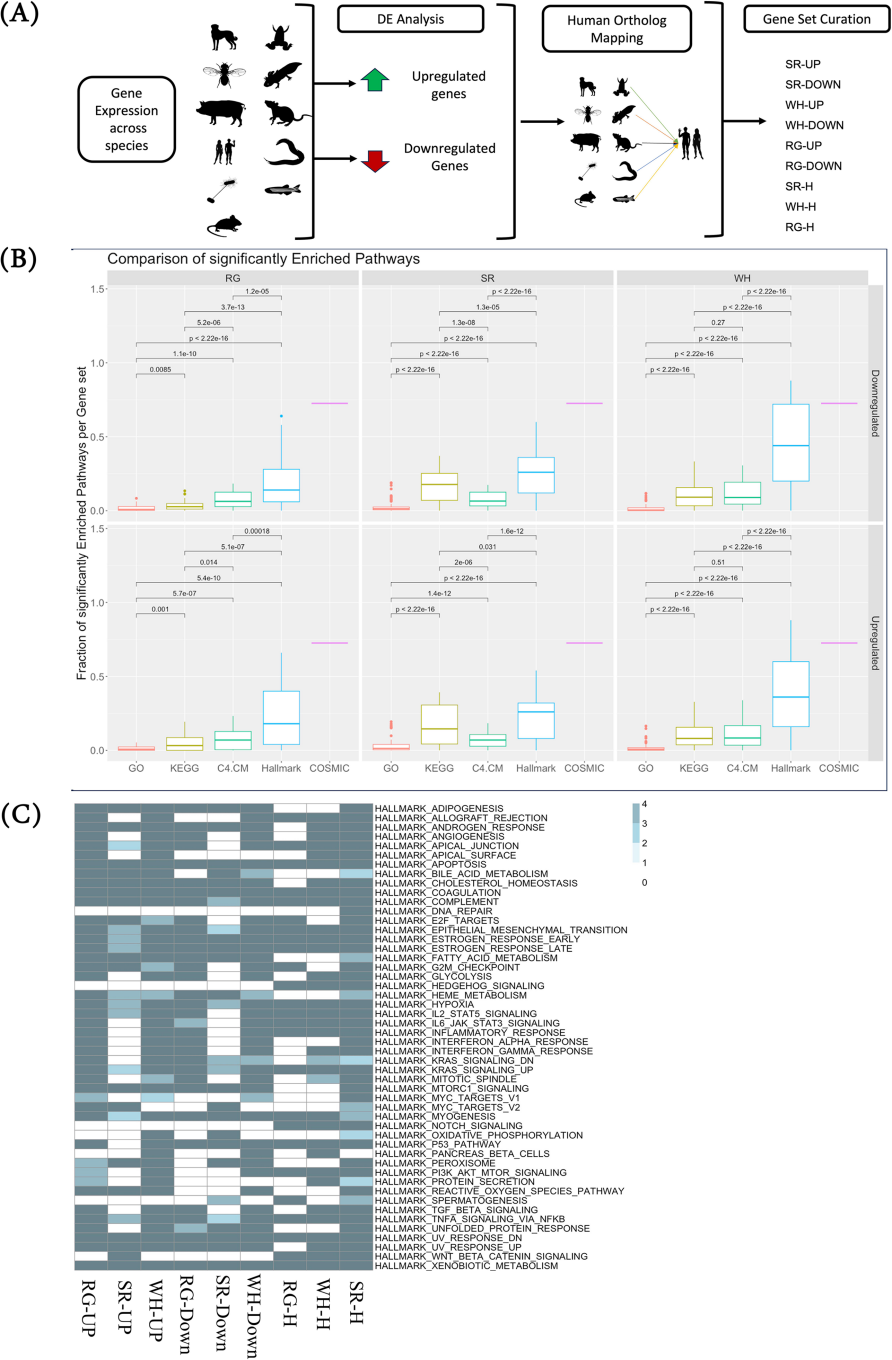
SWR+ gene sets are associated with oncogenic processes. **(A)** Overview of the data curation (see text). **(B)** SWR+ gene sets significantly overlap oncogenic processes; the three columns of the panel correspond to distinct homeostatic processes, with the first row of the panel including upregulated gene sets and the second row including downregulated gene sets. Within each panel, for each gene set X in a collection, the fraction of gene sets in the SWR collection corresponding to the panel (for instance, RG upregulated gene sets for the top left panel) that significantly overlap with X is calculated, and the distribution of these fractions for all gene sets in the collection (x-axis) is shown along the y-axis. COSMIC refers to a single gene set, and hence, a single fraction is shown along the y-axis. **(C)** Cancer hallmark processes are enriched among the SWR+ gene sets. We performed enrichment analysis between the SWR+ gene sets and the oncogenic pathways in the hallmark dataset. All comparisons with significant enrichment (Adj p-value < 0.05) are colored, and the color shade indicates the enrichment odds ratio. SWR, stress–wound–regeneration.

In addition to the pan-species SWR gene sets curated from GEO, we utilized keyword searches in human-centric databases, including GO (23), KEGG (27, 28), and MSigDB (29), to compile three human-specific consensus gene sets—SR-H, WH-H, and RG-H (**Figure 1A**). Note that these gene sets obtained from the human databases are agnostic to the direction of gene expression change. These three human-specific gene sets, combined with the six pan-species experiment-based SWR gene sets, are referred to as SWR+ (**Supplementary Table S2**), which provides the foundation for our subsequent analyses in this study.

### SR, WH, and RG genes are strongly associated with cancer

Here, we investigated the associations of the SWR+ gene sets with oncogenic processes. First, we observed a significant degree of overlap among the nine SWR+ gene sets (**Supplementary Figure S1A**). To investigate the association of the SWR+ gene sets with oncogenic processes, we constructed two collections of oncogenic gene sets: i) the cancer hallmark gene sets comprising 50 gene sets and ii) the MSigDB-C4-CM gene set collection comprising 431 gene sets. For comparison, we used collections of iii) all GO biological processes and iv) all KEGG pathways. For each gene set G in these four collections, using Fisher’s exact test, we calculated the fraction of SWR+ gene sets that significantly (p-value ≤ 0.05) overlapped G. **Figure 1B** shows the distributions of the fractions of significant overlaps for the four collections of gene sets (Hallmark, MSigDB, GO, and KEGG), revealing a significant overlap between the SWR+ gene sets and oncogenic processes, far greater than the overlap with control GO and KEGG processes. Significant overlap of genes (up- and downregulated) holds for species-specific SWR gene sets as well (**Supplementary Figure S1B, C**). Further scrutinizing the highly overlapping gene sets, we found that relative to SR genes, the RG and WH gene sets were significantly enriched for angiogenesis, epithelial–mesenchymal transition (EMT), myogenesis, and oncogenic signaling pathways such as IL6 and TNF. In contrast, the SR gene sets were enriched for MYC Targets_V2, metabolic pathways, and other signaling pathways (**Figure 1C**). Notably, we observed common enrichments across all three homeostatic processes for estrogen response, androgen response, coagulation, KRAS signaling-UP, hypoxia response, and apoptosis. A comparison of the nine SWR+ gene sets with a list of known cancer drivers in the COSMIC database also revealed significant enrichment for cancer drivers (**Figure 1B**).

**Figure 2:**
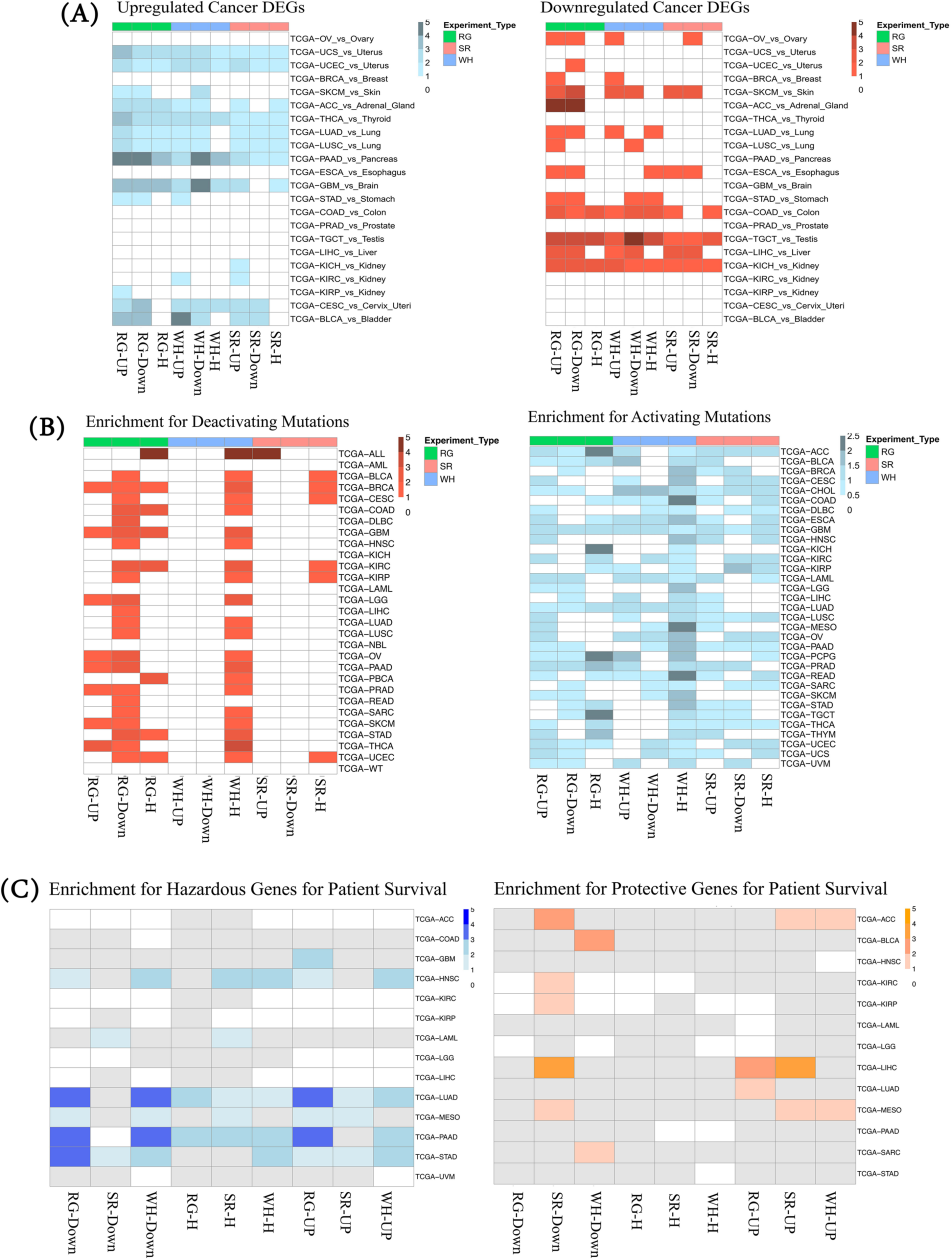
Functional association of SWR+ gene sets with homeostatic processes. **(A)** SWR+ genes are enriched for DEGs across cancers. We performed enrichment analysis between the SWR+ gene sets (column) and the top 10% most differentially expressed genes in each of the 22 TCGA cancer types (row). The heat plots show the significant enrichment (Adj p-value ≤ 0.05) with the color shade indicating the magnitude of the odds ratio. **(B)** SWR+ genes are enriched in activating and deactivating mutations. The figure shows enrichment between SWR+ gene sets and genes that are frequently activated or deactivated (top 5% most frequent) in each of the 33 cancer types. The left panel is for deactivating mutations, and the right panel is for activating mutations. The colored values represent the magnitude of the odds ratio for significant enrichments. **(C)** SWR+ gene sets are enriched for genes associated with patient survival. Based on genes with significantly positive and negative hazard ratios (Methods) in each of the cancer types, the heat plot shows for each SWR+ gene set and cancer type the enrichment in the SWR+ gene set for hazardous genes (left) and protective genes (right) using Fisher’s exact test. The color shade indicates the magnitude of the odds ratio for significant enrichments. SWR, stress–wound–regeneration; DEGs, differentially expressed genes; TCGA, The Cancer Genome Atlas.

Next, we assessed the extent to which the SWR+ genes are differentially expressed in tumors compared to healthy tissues. Toward this, we obtained gene expression data from GTEx (40) and data generated by the TCGA Research Network: https://www.cancer.gov/tcga, and we performed a tumor-vs-normal differential expression analysis in 22 different tissues for which data exist in both TCGA and GTEx (Methods). Using Fisher’s test, we evaluated the enrichment of the nine SWR+ gene sets among genes that were significantly differentially expressed (q-value < 0.05) in cancer. As shown in **Figure 2A**, we observed a consistent enrichment of SWR+ gene sets among DEGs in several cancers. Interestingly, the pattern of enrichment among differentially upregulated genes in cancer was somewhat complementary to that for downregulated genes. Specifically, while tumor-upregulated genes were enriched for SWR+ genes in thyroid, adrenal, lung, bladder, brain, pancreatic, and uterine cancers, tumor-downregulated genes were enriched in colon, skin, testicular, liver, kidney, and stomach cancers. The reasons underlying this heterogeneity are immediately clear and will require further investigation.

Similar to the DEG analysis above, we next assessed enrichment among the SWR+ gene sets for most mutated genes across cancers (**Figure 2B**). Using the TCGA database, across 33 cancer types, we obtained the top 5% most mutated genes based separately on deactivating (including non-sense, out-of-frame indels, and CNV deletions) and activating (CNV amplifications) mutations, and we conducted the enrichment analysis against the SWR+ gene sets separately for each group. Overall, we observed a broad enrichment of activating mutations across SWR+ gene sets and cancer types, while enrichment of deactivating mutations was restricted to regeneration gene sets and in the WH-H gene set. We observed some complementarity where RG-UP tends to be more enriched for activating mutations while RG-Down tends to be more enriched for deactivating mutations, suggesting a link between the activation of regenerative processes and oncogenesis.

Finally, we assessed the association between the SWR+ genes and patient survival in various cancers. Using the Cox proportional hazards model across different cancer types, we identified the genes significantly associated with survival; divided them into two categories, i) hazardous (HR > 1, FDR < 0.05) and ii) protective (HR < 1, FDR < 0.05); and assessed the enrichment of SWR+ gene sets in these two gene sets. We only considered the cancer types with at least 50 significant genes with positive as well as negative HR. Overall, we observed (**Figure 2C**) a strong link between SWR+ gene sets (RG and WH in particular) and poor prognosis (positive hazard ratio) in several cancer types, such as PAAD, STAD, LUAD, HNSC, and MESO. However, we observed broad links between SWR+ gene sets [specifically stress and wound healing genes (up and down)] and with better prognosis (negative hazard ratio) only for the ACC, LIHC, and MESO cancer types. Interestingly, we observed that RG and WH gene sets show significantly greater enrichment for oncogenic processes compared to the SR gene sets. As the gene set size is modest and unlikely to solely account for the observed patterns, the greater enrichment for oncogenic processes in the RG and WH gene sets compared to the SR gene sets likely reflects underlying biological differences.

Overall, these results reveal a substantial functional association of homeostatic processes with cancer in terms of gene overlap, differential expression, mutations, and patient survival. At the same time, these links show variability across cancer types and across the three homeostatic processes. However, a meaningful interpretation of the specific patterns of enrichments (**Figure 2**) is difficult based on our current understanding of the biology of various cancer types and will require further investigation.

### SWR+ genes are proximal to each other and to cancer hallmarks in protein interaction networks

**Figure 3:**
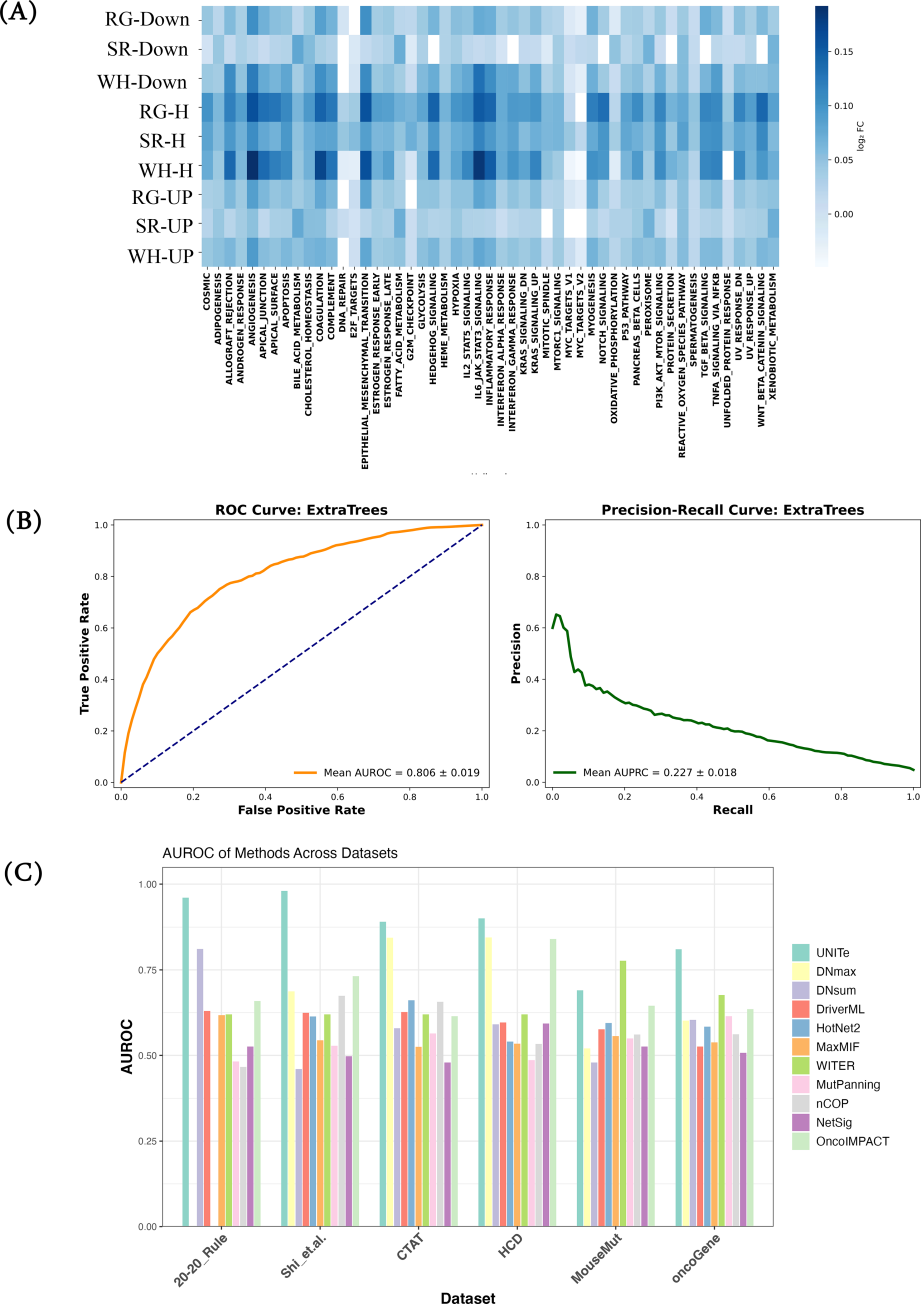
SWR+ is network-proximal to cancer hallmarks and cancer drivers. **(A)** SWR+ is network-proximal to cancer hallmark processes. Here, we calculated the shortest path lengths in the STRING network between each gene in the SWR+ gene sets and the genes in MSigDB (top) and CancerSEA (bottom) gene sets, and we compared the distribution of shortest path lengths with degree-matched controls for each SWR+ gene set. We first compared the distributions of experimental vs. degree-matched control using a Wilcoxon rank-sum test. Heat plot shows the comparisons in blue where the actual distribution is significantly lower than the control, and the shade represents the magnitude of log fold-change between mean of the experimental and matched control sets (lower fold-change is more significant). **(B)** Proximity to SWR+ can be used to distinguish cancer drivers. Here, we used a gradient boosting classifier to train a model to identify known cancer drivers based on the network proximity in STRING network of a gene from various SWR+ gene sets. The figure shows the cross-validation ROC and PRC plots. **(C)** Our model UNITe performs as well as or better than current methods in predicting cancer drivers across multiple benchmarks. Using six independent benchmark datasets (Methods), we evaluated the performance of our model using the area under the receiving operating characteristic (AUROC) and compared it to scores obtained from 10 different cancer driver predictor algorithms. SWR, stress–wound–regeneration; ROC, receiving operating characteristic; UNITe, Uncovering Network-based Interactions between Homeostatic processes and Tumorigenesis.

Proximity in the protein networks has been extensively used as an indicator of functional relatedness between genes (41). Here, we quantified the extent to which the SWR+ genes interact with the oncogenic process in a knowledge-based gene interaction network, STRING, comprising 261,624 interactions between 19,344 proteins. First, we compiled 50 gene sets representing cancer hallmark processes from MSigDB (29), listed in **Supplementary Table S3**. For each of the nine SWR+ gene sets, say X, and each of the 50 hallmark gene sets, say Y, we tested if genes in X were more proximal to genes in Y compared to a degree-controlled background gene set created for each of the nine SWR+ gene sets (Methods). As shown in **Figure 3A**, with the exception of downregulated SR genes, all SWR+ gene sets are significantly proximal to the hallmark gene sets (Methods).

Next, we extended the above analysis to assess the proximity of SWR+ gene sets from known 723 cancer driver genes compiled from the COSMIC database (32). As a control gene set, we created random non-driver genes selected to match the degree distribution (within ±1 degree if needed) of the drivers. **Figure 3A** compares, for each SWR+ gene set, its proximity distribution (Methods) from the driver genes and the degree-matched control. It is evident that all SWR+ gene sets are significantly proximal to the driver genes.

### Network proximity-based prediction of cancer drivers

Given the network proximity of oncogenes from the SWR+ gene sets, here, we assessed the extent to which the network proximity of a gene from the SWR+ gene sets could distinguish oncogenes from other genes. If so, such a prediction model would provide a means to prioritize additional cancer-related genes. Given a gene *g*, we defined nine features relative to the STRING network, where each feature represents, for each of the nine SWR+ gene sets *S*, the fraction of genes in *S* that are within a predetermined distance threshold *d* (≤2) from *g*. We used *d* ≤ 2 for STRING, as “2” corresponded to the median pairwise shortest path lengths in the network. Next, we assessed the prediction accuracy of six different machine learning models (SVM, RF, AdaBoost, Gradient Boosting, Logistic Regression, and ExtraTrees) on nine STRING-based features in a fivefold cross-validation setting. We trained models for different parameters using GridSearch and post-hyper-tuning and optimization (see **Supplementary Table S4**). We found that the ExtraTrees model developed using STRING-based features performs the best (**Figure 3B**; **Supplementary Figure S2A, B**) with an AUROC of 0.81 and an AUPRC of 0.23 (expectation based on positive and negative sample sizes is 0.05), and we used this model for further validations. We used DeLong’s test to directly estimate the superiority of the performance of the ExtraTrees classifier over other classifiers. DeLong’s test is a non-parametric test routinely applied to compare the area under the curve (AUC) of two or more correlated ROC curves, often used in ML studies to determine if one model significantly outperforms another. In our case, the ExtraTrees classifier was significantly better than all other evaluated classifiers: SVM (p = 7.35 × 10^−269^), Random Forest (p = 3.22 × 10^−22^), AdaBoost (p = 5.70 × 10^−85^), Gradient Boosting (p = 4.09 × 10^−45^), and Logistic Regression (p = 7.51 × 10^−62^). Another reason for the better performance of the ExtraTrees classifier over others is its ability to capture the non-linear relationship without requiring extensive hyperparameter tuning. It utilizes fully randomized split selection and ensemble averaging, which effectively reduces variance and model overfitting, leading to both better performance and higher stability across cross-validation folds.

We observed that a potential limitation associated with the current ML models is the class imbalance in the datasets, which is clearly reflected by the AUPRC values. AUPRC values were found to be low and similar across models. One possible explanation of this observation is the tendency of the precision–recall metrics to yield higher false positives, especially at higher recall values, leading to lower absolute AUPRC values even for the models having high discriminative performance as captured by AUROC values.

Shi et al. (37) previously reported a comprehensive benchmarking of multiple methods to identify cancer-related genes across multiple benchmark datasets (Methods). Next, we trained a STRING-based model (that we refer to as UNITe) using all 723 oncogenes from the COSMIC database and degree-matched non-oncogenes, and we assessed its accuracy on six independent benchmark sets in comparison with 10 previously published tools reported in Shi et al. (Methods). In **Figure 3C**, we compare the AUROC achieved using UNITe on these six benchmark sets with the AUROC values for the other 10 tools reported in Shi et al. As can be observed, across all benchmark sets, UNITe outperforms the other tools in distinguishing the oncogenes from non-oncogenes.

### Identification and validation of novel cancer driver candidates

Having established the accuracy of a network proximity-based approach to distinguish cancer drivers, here, we assessed its utility in identifying novel cancer drivers. We created a consensus gold set of 396 cancer driver genes (**Supplementary Table S5**) by compiling genes that appeared in at least two of the following publicly available datasets: Cancer Gene Census (42), OncoGene (43), CTAT (44), 20/20 Rule (45), MouseMut (46), and HCD (47). We selected these benchmark datasets because they were either manually curated or defined using mutational frequency-based models of cancer driver prediction, thus making them independent of network information. We then trained an ExtraTrees model (we refer to this as UNITe) to distinguish the consensus gold set and, as a negative control, degree-matched genes (selected among the genes that did not appear in any benchmark set).

Using the trained UNITe model, we scored every gene in the genome and derived two gene sets: i) UNITe-High: top 2,000 UNITe highest-scoring genes (**Supplementary Table S5**) not included in any of the benchmark sets above or in the SWR+ set; ii) UNITe-Low: bottom 2,000 UNITe lowest-scoring genes (**Supplementary Table S5**) not included in any of the benchmark sets above or in the SWR+ set. Next, we compared UNITe-High with UNITe-Low as well as the Goldset in terms of several expected cancer-associated features utilized above; i) differentially expressed genes (three features: overall, upregulated, and downregulated), ii) genes with high dependency score in cancer type-specific cell lines, iii) genes with significant association with survival (two features: significant positive HR and significant negative HR), and iv) highly mutated genes (Methods). For each of these features, we computed in each of the three gene sets (UNITe-High, UNITe-Low, and Goldset) the fractions of genes having the specific feature, e.g., fraction differentially expressed in cancer relative to normal, and computed the ratio of this fraction for i) UNITe-High with that for UNITe-Low, to assess the biological significance of UNITe-predicted putative cancer drivers, and iii) Goldset with that for UNITe-High (Methods). A positive log-ratio suggests a greater concordance with cancer-associated features.

**Figure 4:**
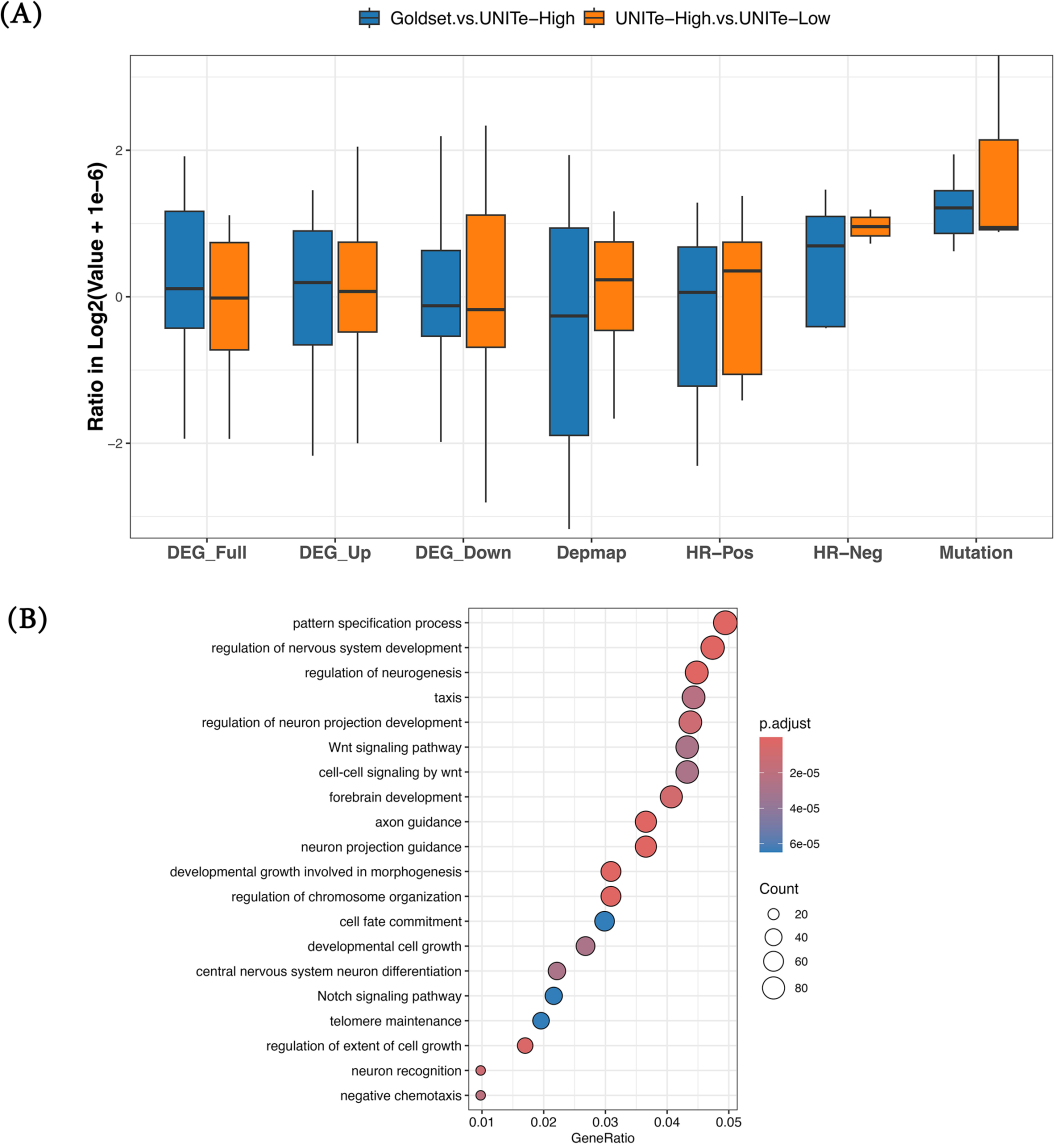
Functional analyses of UNITe-predicted candidate cancer driver genes. **(A)** UNITe-High genes exhibit greater cancer-like attributes in comparison to UNITe-Low genes across cancer types and multiple modalities. The orange boxplots show for each feature (x-axis) the log ratio (y-axis) of i) the fraction of UNITe-High genes having certain feature (x-axis) to ii) the fraction of UNITe-Low genes having the same feature. The blue boxplots show the same comparison of Goldset genes with UNITe-High genes. The features include most differentially expressed (first three plots from left), genes with high dependency score (fourth plot), most associated with survival (fifth and sixth plots), and most frequently mutated (seventh plot). **(B)** Top 20 significantly enriched biological processes associated with predicted top 2,000 UNITe-High genes. UNITe, Uncovering Network-based Interactions between Homeostatic processes and Tumorigenesis.

As shown in **Figure 4A**, for all but one feature, UNITe-High includes a greater fraction of genes having the cancer-associated feature than the UNITe-Low gene set. Goldset is expected to outperform UNITe-High. However, in terms of mutation rates, UNITe-High compares favorably to Goldset. Functional enrichment analysis (**Figure 4B**) among the UNITe-High genes revealed cancer-related processes such as Wnt/Notch signaling, telomere maintenance, regulation of chromosome organization, and neuro-developmental processes, whose roles in oncogenesis have been established (48–50). For example, our model predicted *CSMD3* as a cancer gene with a high score. *CSMD3* encodes for (CUB and Sushi Multiple Domains 3) protein, and as per previous studies, it acts as a tumor suppressor, with a high rate of mutations observed in several cancers, including ovarian (51), colorectal (52), and non-small lung cancers (53). Overall, leveraging network proximity to SWR+ gene sets, UNITe identifies new potential cancer driver genes. **Supplementary Table S5** lists the UNITe-High genes.

## Discussion

Cancer is characterized by breakdown or misappropriation of fundamental homeostatic processes such as stress response (54), wound healing (5), and regeneration (18, 55). Many of the same signaling pathways and molecular players involved in these homeostatic processes, such as inflammation, cell proliferation, migration, and angiogenesis, are also implicated in oncogenesis. While cellular stress response is a key adaptive mechanism for homeostasis, the chronic activation of these responses contributes to cancer development (54). This understanding of the overlapping molecular mechanisms between these processes and cancer could provide valuable insights into cancer biology and potential therapeutic targets.

Functional annotation of human genes is very far from complete, and in the absence of detailed experimental data, various evolutionary and systems-level features have been used to draw functional links between genes and thus ascribe functions to genes with hitherto unknown function following the principle of “guilt by association” (56). One such systems-level feature that has widely been used is physical or inferred functional interactions between genes recorded in the STRING database (35). It has been widely reported that proximity between two genes in protein interaction networks is indicative of their functional relationship. This broad trend has been previously exploited in numerous contexts (57–60) to draw functional links between biological entities, which would be otherwise not feasible at scale.

Following this general principle, here, we investigated links between the three homeostatic processes—SR, WH, and RG—and human cancer. We showed quantitatively, for the first time, that not only are the three homeostatic processes proximal to each other and to cancer driver genes in the protein interaction network, but the proximity of an arbitrary gene from the genes involved in these three processes can distinguish cancer drivers more accurately than multiple previous approaches against multiple benchmark cancer driver datasets. The accuracy of our approach, given its simplicity, is noteworthy. Our tool helps identify several novel candidates for cancer drivers that exhibit several characteristics of known cancer drivers, such as differential upregulation in tumor compared to normal tissue, higher gene dependency scores, and enrichment for frequently mutated genes in cancer.

Another contribution of our work is a resource of curated genes experimentally shown to be involved in SR, WH, and RG across 75 studies spanning 321 experiments across 14 species. Despite the fact that the genes were observed in experiments conducted in highly diverged species, their human orthologs are i) enriched for numerous oncogenic processes, ii) differentially expressed across many human cancers, and iii) enriched for cancer-associated mutations.

One of the limitations of our study is that the curated SWR genes, by virtue of being obtained from experimental data in other species, lack the tissue context. The protein interaction network that we and others use is also context-agnostic. Accordingly, our study looks at the relationships between SWR genes and a pan-context set of cancer drivers, and likewise, our tool predicts cancer drivers in a context-agnostic fashion. Context-specific gene functionality is largely unknown, and computational approaches have been proposed to infer the context-specific function of genes (61–63). Approaches to project the context-agnostic protein network to a specific tissue context based on the tissue-specific transcriptome have been proposed (62). In the future, it would be worthwhile to apply the tissue-specific networks to prioritize tissue-specific cancer drivers.

## Conclusions

Overall, our study shows that three conserved homeostatic processes—stress response, wound healing, and regeneration—are closely linked to human cancer. Using protein interaction networks, we quantified these links in terms of network proximities and introduced a simple network-based approach to prioritize cancer driver genes, with the potential to extend this framework to tissue-specific cancers. We also provided a curated cross-species gene resource for the three homeostatic processes and presented a machine learning model, UNITe, that can identify novel cancer driver genes.

## Data Availability

The original contributions presented in the study are included in the article/**Supplementary Material**. Further inquiries can be directed to the corresponding author.
