# Supplementary material for: UNITe: exploiting conserved links between oncogenesis and homeostasis to identify novel cancer drivers
Source: Front Oncol. 2026 Feb 24;16:1711517. doi: 10.3389/fonc.2026.1711517 (PMC12971524; doi:10.3389/fonc.2026.1711517)
Supplement: Supplementary file 1 [file DataSheet1.docx]

**UNITe: Exploiting Conserved links between Oncogenesis and Homeostasis to Identify Novel Cancer Drivers**

Piyush Agrawal^1^, Annan Timon^2^, Vishaka Gopalan^3^, Arashdeep Singh^3^, Sridhar Hannenhalli^3^*

1. Division of Medical Research, SRM Medical College Hospital & Research Centre, SRMIST, Kattankulathur, Chennai, India
2. University of Pennsylvania, Philadelphia, PA, USA
3. Cancer Data Science Lab, National Cancer Institute, NIH, Bethesda, MD, USA

*Corresponding author: sridhar.hannenhalli@nih.gov

**Supplemental Figure S1 A.** Upset plot representing the degree of overlap among the SWR+  gene sets.

**Supplemental Figure S1B.** Upset plot showing the overlap of upregulated genes for species-specific SWR gene sets as well.

**Supplemental Figure S1C.** Upset plot showing the overlap of downregulated genes for species-specific SWR gene sets as well.

**Supplementary Figure S2A. Proximity to SWR+ can be used to distinguish cancer drivers:** The figure shows the cross-validation ROC plots. Here, we use a 5 different classifier to train a model to identify known cancer drivers based on the network-proximity in STRING network of a gene from various SWR+ gene sets.

**Supplementary Figure S2B. Proximity to SWR+ can be used to distinguish cancer drivers:** The figure shows the cross-validation precision-recall plots. Here, we use a 5 different classifier to train a model to identify known cancer drivers based on the network-proximity in STRING network of a gene from various SWR+ gene sets.
